# The relationship between social life and emotions. Adam Ferguson and sociology

**DOI:** 10.3389/fsoc.2023.1194280

**Published:** 2023-06-05

**Authors:** Emiliano Bevilacqua

**Affiliations:** Department of Human and Social Sciences, University of Salento, Lecce, Italy

**Keywords:** emotion, sociology, modernity, Ferguson, Scottish Enlightenment

## Abstract

Adam Ferguson has a leading position among those who have developed a sociological interpretation of modernity that dismiss metaphysics without following the echoes of rationalism. Ferguson outlines a vision of social life that correlates the analysis of individual behavior to the study of social context and institutions. Consistently with this approach, the Scottish scholar emphasizes the multidimensionality of human beings without forgetting the non-rational features of social behavior. This essay aims to discuss Ferguson's thought with a special attention to the importance of the emotions in social life, so as to enhance the contribution of classical sociology to the analysis of the emotionality. Ferguson, in fact, argues that emotions have a leading role in shaping the behaviors and values of individuals. Developed in the context of Scottish Enlightenment, Ferguson's sociology shows how the study of modern society can be reconciled with a reasonable as well as emotional approach to social life.

## 1. Introduction

In the history of social thought, the debate over the origin of disciplines has often highlighted how the variability of epistemologies influences the order of priorities and interests characterizing different historical moments. By emphasizing certain topics rather than others, each historical-disciplinary configuration retrospectively illuminates its past whilst revealing a new sensibility (Barnes et al., 1986). The emotional turn in sociology, from this point of view, shows a growing dissatisfaction for the solutions that this discipline has advanced as a result of the difficulties encountered by rationalistic approaches in explaining social behavior (Turner and Stets, 2005).

The Scottish Enlightenment represents an already fully proto-sociological field of investigation, whose significance for sociology acquires greater importance if one evaluates more carefully the role that emotions play in it. Emotions fuel social behaviors and, consequently, enrich our understanding of society. Social research over the past century has either sacrificed the study of emotionality on the altar of a utilitarian interpretation of rationality or privileged a structuralist view of the social order in which individuals' emotional tendencies played no role. More recently, however, sociology has progressively understood how emotions and reason are not antithetical but complementary (Turner and Stets, 2005). The former, in fact, are helpful to the latter as they allow for a better subjective understanding of society, as long as the world around us is a universe made up of social interactions that emotions allow individuals to manage more effectively (Damasio, 1994). Furthermore, emotions influence morality as long as affective actions support the stabilization of collective behaviors and their institutionalization, thereby promoting the reproduction of shared values (McKenzie, 2019). Even the phenomenon of internalizing social values through the process of socialization reveals an important emotional dimension (Reynolds, 1993, 2003). Although the impact of emotions on social life can lead to controversial situations, social behaviors, and collective beliefs show an emotional dimension that Ferguson in his pioneering sociological work, fully enhance.

This essay shows how Adam Ferguson uses emotions as analytical tools serving social theory, by deploying their heuristic potential within an overall sociological framework. Ferguson, in fact, develops an interpretation of social life that considers the subjectivation processes by correlating them with the study of social processes leading to commercial society and modernity. His profile is that of a classical author aware of the variety of individual motivations fueling social behaviors and shaping moral values (Lehmann, 1930).

His consideration of the emotions as determining variables for his analysis constitutes a particularly timely element in the discussion on the motivations at the basis of the social action that animates sociology. Being aware that the scientific discussion on authors is a precondition for the analysis of the most up-to-date social phenomena (Baert, 2013; Sapiro et al., 2020), this essay aims to discuss Ferguson's thought so as to enhance the contribution of classical sociology to the analysis of the emotions (Cerulo and Scribano, 2022). The inquiry on the theoretical foundations of sociology is an important contribution to the growing interest toward the role played by emotions in social life. First of all, it is important to deal with the Scottish Enlightenment so as to highlight how Adam Ferguson's more immediately sociological elements exemplify a broader research context.

## 2. The Scottish Enlightenment's legacy

Scottish Enlightenment authors assign social life a crucial role in understanding institutional patterns and individual behavior and values (Broadie, 2003; Brewer, 2014). They proceed in two directions: on one hand, moving away from philosophical speculation to focus on social relations study, and downgrading the rational dimension of human action to open up to a consideration of the emotions, on the other. The Scottish Enlightenment sketches a sociology in the making that anticipates today's interest to the emotional aspects of social life.

The shift brought about by the Enlightenment is emphasized by the sociologist Alan Swingewood when he argues that “[…] a commitment to historical and scientific modes of thought and inquired shifted the prevailing discourse of political and moral philosophy away from traditional concerns with the universal and the transhistorical to a grasp of the specificity of the social” (Swingewood, 2000, p. 3); but in the second half of the 18^th^ Century it was the Scottish Enlightenment that linked the study of transformation with the interaction between social structure and individual behavior, so that “[T]he writers of the Scottish Enlightenment developed a core of sociological concepts and an empirical methodology subsisting with economic, political and historical perspectives” (Swingewood, 2000, p. 7; also Swingewood, 1970).

Swingewood argues that it is a historic limitation of the sociological thought that the achievements of Adam Ferguson, John Millar and Adam Smith were lost during the 20^th^ Century. Already in the 1960s, after all, British sociology had pointed out how “[T]he Scottish moralists are men whose work in a variety of area included much than today we would unhesitatingly call ‘sociological”' (Schneider, 1967, p. 14), underlying how “[F]or authentic theories of social evolution before Darwin one can turn nowhere better than to 18^th^ century Scotland. There is no greater gap in the history of the social sciences than the lack of any adequate account or appraisal of the comparative investigation of institutions by such men as Ferguson and Millar, Monboddo, Kames and their associates” (MacRae, 1961, p. 138).

The Scottish authors' interest in social relations was highlighted by David Riesman in showing how Adam Smith anticipated the Durkheimian critique of utilitarian interpretations of the individual behavior. The American sociologist points out how Smith believes that the subjectively attributed meaning of human actions is essential to understand social interaction. He, also, recalls how this approach, in addition to foreshadowing the Max Weber's theory of action, moves Smith's analysis away from political economy and toward the idea that individual behavior is dependent on “a group mind” defining the specific field of sociology. Because “[…] both Smith and Durkheim held that collective beliefs and practices are found in each individual precisely because they exist in the group, and that the process of socialization internalizes such beliefs and practices in the form of rules” (Riesman, 1976, p. 70), follows that adherence to social norms, rather than being caused by an economically motivated utilitarian association, derives essentially from the “love of community” (Riesman, 1976, p. 67; also Trigilia, 1998).

These considerations offer a good exemplification of the Scottish tendency to abandon a deterministic approach to the study of human nature to focus on contextualized social interaction in seeking to identify the ideal orientations and collective behaviors that structure society. Thus, the sociological consequentiality of the Scottish Enlightenment is not surprising. Based on an immanent analysis of social processes, the Scottish Enlightenment pays particular attention to the role played by the emotions in social life, thus rejecting a fideistic adherence to the principle of rationality: “[I]n Scotland (most explicitly in the case of Hume) there was a turning away from rationalism toward modesty as to the scope for establishing certain knowledge. By embracing the understanding of rationalism as a dead end, the Scottish philosophers turned instead to building a science of human nature as the foundation of all other scientific knowledge. Reason would come only after belief, sentiment and experience” (Dow and Dow, 2006, p. 3). Furthermore, Alexander Dow and Sheila Dow retrace the history of Scottish economic thought by highlighting how one of the hallmarks of this social theory was “[T]he specification of first principles in terms of a non-individualistic representation of human nature, with a consequent emphasis on conventional behavior” (Dow and Dow, 2006, p. 4).

The Scottish Enlightenment's pioneering focus on sociality is coupled with a multidimensional view of subjectivity, including the role of emotions in social life. This sociological approach has three dimensions. Firstly, the Scottish Enlightenment attributed scientific autonomy to social processes as opposed to morality and law; secondly, they showed how social order was the consequence of institutions in harmony with social interactions shaping modernity; at last, this school believed that individual and collective wellbeing was based on an multidimensional and emotional subjectivity, quite distant from the one-sided representations of pre-modern thought. The focus on the generative processes of the social shapes a vision in which the emotional dimension plays an analytically relevant role.

The historical contextualization of social processes, beginning with David Hume, is decisive for scholars of the Scottish Enlightenment so that the theories of the state of nature are progressively subjected to a particularly sharp critical scrutiny (Berry, 1997). Hutchison (1988, p. 332–358), for instance, points out how the successors of Humean philosophy, among whom he includes Ferguson, Millar and Smith, as well as James Stuart, develop his teachings precisely on the ground of social theory. Based on this interpretation, they reject the contractualistic view of social relations both as encumbered with a flaw of a-historicalness and as an expression of a pessimistic and rationalistic as well as metaphysical human vision. This does not imply that human behavior is without purpose but simply that social institutions cannot be explained as the inevitable outcome of a consciously directed human design, whether contractualistic or otherwise: thus, the famous Fergusonian sentence for which “[E]very step and every movement of the multitude, even in what are termed enlightened ages, are made with equal blindness to the future; and nations stumble upon establishments, which are indeed the result of human action, but not the execution of any human design” (Ferguson, 1819, p. 222) suggests the importance of both the socio-historical variability of human institutions and the complexity of individual orientations.

The Scottish Enlightenment's focus on society concludes in the claim of social interdependence as a central factor in understanding social processes. Rejecting those interpretations that explain commercial society as an inevitable consequence of a divine plan, or an equally inescapable outcome of a spontaneous and utilitarian convergence of interests (Berry, 2013), civil society emerges as a reference for the development of a theory that claims the autonomy of social dimension in the interpretation of modernity. If for the Scottish Enlightenment pioneer Francis Hutcheson, the main feature of the human species is the natural propensity to social life (Hutcheson, 1726 [2008]; cf. also Zanini, 2014), the individual character and the model of society will develop a mutual dependence that can be observed mainly focusing on social interaction (Ahnert and Manning, 2011).

Moreover, the social order is seen as an institutional equilibrium capable of integrating the needs of the community with individual wellbeing deriving from this: “[I]t is one of the hallmarks of the Scottish Enlightenment that (to use later terminology) it recognizes both structure and agency” (Berry, 1997, p. 42). The sociological profile of this perspective emerges with particular strength when looking more closely at the way in which these authors discussed the problems of integration posed to society by the impetuous development of the market economy. In this way, the analytical starting point depends on the study of social processes and concludes in an awareness of an equilibrium that balances the stability of the new economic order with the freedom and complexity of human needs. The discussion of commerce pioneered by Hume (1994), for example, highlights an approach based on the possibility to increase a virtuous circle between the greatness of the state deriving from the wealth generated by the interchange of goods and services, on the one hand, and the economic satisfaction and social recognition of the subjective aspirations poured out by individuals in the market, on the other. This analysis conveys an idea of social order strongly centered on individual fulfillment achieved in a relational context. The same Smithian interpretation of the subjective motivations driving economic action shows how professional and work activities are supported by sentimental reasons (Rothschild, 2001), linked both to an individual inclination to convey the need for sociality in the commercial sphere (Smith, 1978) and to the conviction that the consideration of others can be achieved by economic activity (Smith, 1984).

The reflection over human nature developed by the Scottish Enlightenment is the sociological dimension that more than any other allows us to appreciate the interest shown by these authors in the emotions. Beyond the awareness of personality changes as historical time changes, and beyond the ability to consider the weight of daily interactions in determining important socio-economic phenomena, we find in the Scotland of the second half of the 18^th^ Century we find a special emphasis on the passions and their social relevance.

Singer (2004) precisely linked the emergence of the “social” with the emphasis on the passions when he suggested that Smith, along with Montesquieu, considers emotional communication and its social construction as a pillar of the study of society. But, along with this important acquisition, there is, particularly in authors such as George Croom Robertson or Henry Home (Lord Kames), a further awareness, namely that desire is a relevant social phenomenon and has a social history worthy of attention. The realization, for example, that “desire, for these Scottish Enlightenment authors, was, in part, the product of constructed gender difference” (Moloney, 2005, p. 248) offers a particularly timely exemplification of a theoretical current for which the centrality of socialization and feelings, from James Fordyce to the better-known John Millar, also embraces sexual attraction: while it is true that “[A] major insight of the enlightened writers was the primary role which the human emotions played in individual motivation and social organization” (Dwyer, 1998, p. 1), nevertheless “[T]hey focused on the cultural possibilities of love and sexuality. Scottish writers were pioneers in analyzing the ways in which sexual attraction could be modified into a less volatile and more enduring social feeling” (Dwyer, 1998, p. 5).

## 3. Ferguson on sociology

Adam Ferguson has a leading position among those who have developed a sociological interpretation of modernity that dismiss metaphysics without following the echoes of rationalism (Macrae, 1966; 1969; Meek, 1976; Bierstedt, 1979; Barbalet, 2004). His critique of contractualism, for instance, expresses mistrust for oversimplified solutions to the problem of social order but avoids concluding in the exclusive claim of entirely utilitarian rational behavior; on the contrary, his reflection on the underlying tendencies of human nature is matched with the observation of the historical variability of social behavior, leaving much space for the influence performed by society upon the complex grounds that constitute subjectivity.

In his *Essay on the History of Civil Society*, Ferguson stigmatizes the state of nature by stating that in it “[…] men are made to unite from principle of affection, or from principle of fear, as is most suitable to the system of different writers” (Ferguson, 1819, p. 21) while “[T]he history of our species indeed abundantly shows, that they are to one another mutual objects both of fear and of love” (Ferguson, 1819, p. 21). From this point of view, does not surprise that David Kettler refers to the *Essay* by mentioning that “[I]t was in this work that he developed the essential elements of his orientation to society” (Kettler, [1965] 2005, p. 201). The Scottish scholar affirms indeed that human nature is intimately sociable but extremely complex and, therefore, devoid of everlasting arrangements, emphasizing how the mutability of attitudes and situations makes impossible the identification of any original state. Mental flexibility and the different inclinations of individuals highlight how the extremes represented by the condition of the savage rather than that of the citizen both express socially possible solutions: “If the palace be unnatural, the cottage is so no less and the highest refinements of political and moral apprehension, are not more artificial in their kind, than the first operations of sentiment and reason” (Ferguson, 1819, p. 14). Furthermore, Ferguson does not merely show how social processes shape human history, but makes a more specific argument in stating that is the cultivation of social ties that enhance individuals' capacities: “[T]hat condition is surely favorable to the nature of any being, in which his force is increased; and if courage be the gift of society to man, we have reason to consider his union with his species as the noblest part of his fortune” (Ferguson, 1819, p. 33). Social groups are acknowledged as catalysts of human forces and passions, hence as aggregates increasing individual expectations, although equal attention is paid to friendship and emotional relationships that bring together two individualities. The latter constitutes the ideal ground for those particular social relations that allow the individual to develop his specific subjectivity and to mature his moral dispositions, which are however always influenced by the relevant social context. Ferguson highlights how social interaction experienced at group as well as dyadic level influences identity by shaping social individuals who, in turn, are affected by the historical context in which they operate, i.e., the conditions formed as a result of the slow accumulation of past social interactions. This approach permeates the whole Fergusonian work.

Ferguson's political philosophy shows a strong dependence on the influence that social dynamics have on individual values and behaviors. We can interpret both the belief that the state that favors citizen autonomy is the most appreciable model of political organization and the assertion that the strength of the nation rests essentially in the character of its citizens as different exemplifications of the same approach to political phenomena. Ferguson's emphasis on civil society demonstrates a view of society and human nature in which institutions derive from the balance between individual autonomy and social conditioning. The history of nations is indeed strongly linked to the character formation of the citizen and to the virtuous circle between the richness of social life and personal growth, as extensively discussed in *Reflection Previous to the Establishment of a Militia* (Ferguson, 1756). Craig Smith, to this regard, argues that “[T]his leads Ferguson to understand the situation of a nation at any given point in its history as a form of balance arising from social interaction. The ‘vigor' of a state is related to Ferguson's theme of liberty secured by balance, competition and an active citizenship and, in turn, this idea of vigor is advanced in the twin languages of healthy individual and national character” (Smith, 2008, p. 166).

Human sociality constitutes a reference point even for Ferguson's explanation of change. Although the Scottish scholar's rejection to consider the emergence of a new social order as the consequence of intentionally directed individual actions has been extensively debated epistemologically and even politically (Hayek, 2014), we direct our attention essentially to the sociological implications of this approach. Ferguson dismisses the existence of deterministic social laws but believes that there are general tendencies acting on both the individual and the collective level. All these tendencies are related to the sociable nature of individuals. Eugene Heat emphasizes how Ferguson attributes the starting conditions of all change to the original presence of roughly differentiated social groups, to the role of the family considered as the primordial social group, and to the set of values and environmental conditions that characterize the different historical moments. It is possible to outline these Fergusonian suggestions by stating that “[T]he law of society would seem to include a fundamental disposition to adhere to the group; a certain natural and unreflective ease in the communication of passions and sentiments; a propensity to imitate others; an ability to learn (in the sense of ‘improve') via ‘example and intercourse'; and a receptivity to encouragement and praise” (Heat, 2009, p. 165). In addition to, from an individual point of view, there is a tendency to improve one's own condition, which results in particularly significant collective behavior: “Thus do we have four law-like tendencies: one to initiate (ambition); one to communicate (society); one to preserve (habit); and one to distinguish and challenge (conflict)” (Heat, 2009, p. 166). The combination of these tendencies outlines an analysis of social change that holds together socio-cultural conditions and individual expectations, confirming Ferguson's reluctance toward metaphysical and one-dimensional theoretical solutions. The rejection of finalism, in this case, favors a vision that place side by side the macro-dimension of social context and their institutions with the micro-dimension of individual behaviors.

This interdependence, for instance, emerges clearly from the way Ferguson transposes the Stoic category of virtue following the interpretative tradition of the Scottish Enlightenment, which is particularly oriented toward the historicization of human nature. Starting from the premise that humans are essentially social beings, the Scottish scholar inscribes virtue into his theory (Kettler, [1965] 2005, pp. 187–316) and underlines how individuals express themselves fully in the development of social relations rather than in the pursuit of immediate utility. The conclusion is that “[V]irtue, after all, is the perfection of human nature, and human nature is inconceivable without society” (Kettler, [1965] 2005, p. 188). In this way, virtue expresses the mature stage of subjectivity and shows as much as the individual desire for betterment and the human inclination toward sociability (Graham, 2013, p. 516–519). The Scottish scholar, therefore, allows the observation of a correspondence between the development of subjectivity and that of the social order to emerge from the consideration of the intense relations that men entertain with each other, such as a convergence between the extension of social relations proper to civil society and the processes of modernization.

Ferguson thus reflects on the social processes that lead Scotland of the late 18^th^ Century toward the decline of classically traditional social groups and the increasing relevance of individual autonomy in social life. He comes to the conclusion that a social model is possible where individuals can develop their multifaceted personalities by cultivating political, cultural but also economic dimensions (Berry, 2009, p. 143–144). The desire for betterment that drives subjectivity does not invest a single dimension of the human being but finds expression in a overall maturation. Similarly, self-realization does not derive from the attainment of a state rather than from the ownership of a good, but follows from the immanence of everyday action in a social context. Indeed, practical experience developed in the course of social life gives meaning to the world and structures one's personality. While it is true that “[H]appiness is attained from the exercise of active pursuit, not from the satisfaction of securing the goal, and as such it is not a static outcome but a dynamic process” (Smith, 2008, p. 161), then “[T]he underlying dichotomy was not between savage and civilized man, but between man employed in action, natural or moral, and man languishing in corruption or slavery” (Oz-Salzberger, 2008, p. 149). It is important to emphasize, in this regard, how the Fergusonian discussion of virtue moves away from Stoicism in order to envisage a modern subjectivity that, in the course of time, is able to express its complexity. Given the fact that this is a social process, does not surprise that the Scottish sociologist develops his analysis of the human nature by assigning particular importance to the category of civil society (Ferrarotti, 1984). Ferguson's thought is directed toward the processes of social integration in commercial society and the possibility that modernity recognizes individual autonomy within a stable and prosperous social order.

This approach expresses a critique of the model of economic man and his instrumental rationalism, but also differs itself from the stoic tradition by basing human nature on a socio-historical analysis of man and his interpersonal context. Ferguson believes that the social order can stabilize and reproduce itself if it provides a liberal regulation of different individual motivations. The virtuous man is, indeed, a well-socialized citizen also positively oriented toward his own personal fulfillment. Ferguson's interest for a possible convergence between happiness and virtue can be seen as the search for a social organization in which there is a relative correspondence among individual and collective needs: “[T]rue happiness is to be found in the active exercise of virtue. As a result, happiness and virtue are identical and both are the product of the proper exercise of our active natures. A consequence of this is that virtue itself is an ‘active' principle in nature, and, moreover, that it is most effectually attained when men are vigorously engaged in serious ‘business”' (Smith, 2008, p. 163). We can thus interpret Ferguson's tendency to temper the conflict between individual and collective interests as a sign of the Scottish scholar's attention to a society able to integrate individuals without neglecting their specificities and autonomy; thus happiness can coincide with the possibility that everyone makes “[…] to make his social dispositions the rating spring of his occupations” (Ferguson, 1819, p. 99) and in feeling part of a community in which values enlighten one's own reason and emotions.

Ferguson share with other scholars of the Scottish Enlightenment the critique to the state of nature as an abstract and astoric explanation of the social order developing an original interpretation of politics seen as expression of civil society. It is not, in this latter case, a claim for the autonomy of the market against the public regulation of social life, but rather the assertion of a decisive correspondence between social processes and political regulation. When the Scottish scholar argues that, “[F]orms of government are supposed to decide of the happiness or misery of mankind” but “[…] forms of government must be varied, in order to suit the extent, the way of subsistence, the character, and the manners of different nations.” (Ferguson, 1819, p. 113), he envisages a sociological analysis that invests the explanation of change, observed as a process of transformation of both habit and personality. Ferguson interprets virtue as a natural expression of the individual as such in his social dimension, inevitably included in the historical context to which it belongs. This leads to restore the sense of a social theory that invests in the complexity of human nature and paves the way for the full appreciation of the emotional dimension that characterizes human beings.

## 4. Emotions and social life

Ferguson's focus on emotions emerges as a consequence of an observation on the relationship between the human tendency toward self-preservation, on the one hand, and the moral education of the individual, on the other. The Scottish scholar remarks that the proper recognition of an instinct of self-preservation that helps humans to preserve life by seeking pleasure and shunning pain is at the basis of utilitarianism. At the same time, he believes that this instinct does not exhaust the whole range of motivations driving social behavior. Although several authors of his time refer to actions aimed at self-love, Ferguson rejects this expression by connoting all these activities in terms of simple self-interest. Indeed, the moral education of the individual brings with it further expectations to those involved in the reproduction of life and the pursuit of pleasure.

The critique of utilitarianism is relevant as long as refusing to use the expression “self-love” to refer to the activities involved shows a particular idea of the social human being, no longer one-dimensional and thus essentially self-centered but, on the contrary, multidimensional in its motivations and open to disinterested social relationships. Ferguson, indeed, believes that human happiness goes beyond the activities of self-protection and self-reproduction because, alongside with the protection and enhancement of the body and property, people reveal a diversity of personal inclinations among which the disinterested passions, i.e., love, tenderness, affection or courage, and have a prominent place.

This vision of life in society assigns a prominent role to emotions since they shape social relations by directing individual behavior. Referring to childhood games rather than adult disputes, Ferguson draws attention to emotions both as dispositions orienting individuals toward happiness and as vectors of social interaction fostering or disfavoring the cohesion of groups and institutions. A narrow and limited pleasure principle, exclusively aimed at self-preservation, is thus insufficient to explain social life and to understand individual action. If it is plausible that in pursuing self-interest individuals neglect other motivations of the human nature, the opposite is often the case: both children and adults lose sight of the needs of self-preservation when they are overwhelmed by the passions of the heart that arise in society. This is evident in the emotional involvement that characterizes childish games, invests everyday sociality or animates the most challenging disputes of values or ideals. If it is true that “[T]he bosom kindles in company, while the point of interest in view has nothing to inflame” because “[…]a matter frivolous in itself, becomes important, when it serves to bring to light the intentions and characters of men” (Ferguson, 1819, p. 58–59), then are the emotions arising from social interaction that shape individual identity and moral values: “[T]o distinguish men by the difference of their moral qualities, to espouse one party from sense of justice, to oppose another even with indignation when excited by iniquity, are the common indications of probity, and the operations of an animated, upright, and generous spirit” (Ferguson, 1819, p. 70).

Furthermore, happiness does not correspond to the achievement of a state, the attainment of a goal or the acquisition of objects but derives from the process that leads to these goals, i.e., the mental and emotional motion finalizing human expectations by placing individuals each other in contact. Events and activities of everyday life structuring social life, therefore, represent a constant opportunity for subjective fulfillment. The activation of reason and emotions experienced by individuals while acting in society offers the opportunity to feel oneself in the world, to express one's inner dispositions, to structure an identity. Ultimately, happiness comes from the ability to make “[...] a certain species of conduct our amusements [...]” and consider “[...] life in the general estimate of its value, as well on every particular occasion, as mere scene for the exercise of the mind, and the engagements of the heart” (Ferguson, 1819, p. 92). While the Scottish scholar considers happiness to be the goal of life, he inevitably shows the emotional profile of human motivations; similarly, the processual nature of happiness calls into question the perception and enhancement of feelings accompanying the physiology of social interaction.

Ferguson understands virtue as a natural expression of the self as a fully social manifestation of each individuality, inevitably included in the historical context to which it belongs. This leads to a complexity of human nature and opens the way to a full appreciation of the emotional dimension that characterizes humans. Emotions play a role in this space of identity shaping, both as an essential dimension of an everchanging nature of the human being and as an important part of our social life. Happiness as a process, moreover, occupies an essential position in Ferguson's social theory because “[A]s Ferguson says, the point of all systems and institutions is to make people happy. This is ‘the fundamental principle of political science”' (Hill, 2006, p. 18).

Individuals therefore, as clearly illustrated in *Institutes of Moral Philosophy* (Ferguson, 1800), actively pursue their inclinations, aiming at self-preservation, but also activating social behavior of cooperation and altruism, as well as cultivating interiority, beauty and art. Lastly, they morally characterize both the interactions they experience in the course of their life and the objects they use or covet. Emotions foster these processes. They forge both individual happiness and the social shaping of moral values.

Ferguson outlines a framework of individual behavior forged on the basis of recurring associations between specific social situations and particular emotional reactions. Everyday life is the investigative ground that allows the Scottish scholar to observe social processes in their immanence. Habits are influenced by emotions because, from a subjective point of view, individuals interact and communicate with each other so as to identify with the feelings of others, while, from a social point of view, emotions tend to propagate and exert their influence through contagion. Lisa Hill, from this point of view, has summarized the ways in which emotions act in social life: “[F]or example, class resentment, covetousness and envy lead to formal government as well as political faction fighting and (therefore, in turn) the preservation of rights and liberties; invidious comparison generates the quest for excellence and the pursuit of wealth and progress; parental affection leads to the formation and maintenance of the family and later the nation-state; shame operates as a powerful means of social control; material and moral progress spring from ‘ambition' which is a combination of restlessness, activism and a compulsive desire for improvement; competition, conflict, belligerence and hostility indirectly preserve social cohesion and give rise to such beneficial institutions as an organized state, positive law and advances in defense technology and statecraft; social norms are shaped by and depend upon inter-subjective validation while moral judgements are reinforced by shared or mutual affective responses” (Hill, 2006, p. 16). Although the broad spectrum of social expressions of the emotions justifies a concern about the risks to the social order that could arise from them, thus threatening the proper conduct of social life, Ferguson's analysis nevertheless emphasizes the integrating effect of the individual manifestation of emotions and their socialization, both for everyday social interaction and the shaping of moral values. Indeed, emotions consolidate collective behaviors by cultivating social morality.

This does not mean that this process is unavoidable since, as we have mentioned, Ferguson believes that society is the outcome of individual behaviors not always purposive. However, it is important to emphasize that social interaction allows a set of values and behaviors to emerge in a progressive, albeit non-linear manner, on which Ferguson believes emotions exert a significative influence. Although “[U]nlike Hume or Smith he has not isolated one specific feature of human psychology that would serve to aggregate or coordinate disparate phenomena” (Heat, 2009, p. 168), the Scottish scholar has shown how “[O]ut of a series of contingencies and law-like processes, an objective set of moral standards emerges” (Heat, 2009, p. 168) offering, beyond any teleological view, an historical record of the social processes driving individuals and shaping values. He interprets the history of civil society as a succession of social stages in which the diversity of social ties and emotions is decisive for the progress and stabilization of the social order (Smith, 2018).

Emotions, moreover, relate to knowledge not only when they compensate wisdom with intuition where reason fails (Ferguson, 1792, p. 77–78), but also when they perform an ordering function with respect to the overabundance of signals that the surrounding environment sends to the individual through the senses. Emotions not only support positive knowledge of the world, but also enable us to better understand our social roles, those of others, and the many inner implications they carry out: “If we overlook the characteristical qualities by which subjects may be distinguished or classed, the world, in respect to us, yet remains in a state of confusion or chaos: If we overlook the more important relations of action and passion, by which parts are combined in the living order of nature, we remain insensible to that magnificent scene which the universe presents, and in the contemplation of which we are defined to find the highest and molt improving exercise of our faculties” (Ferguson, 1792, p. 94).

Besides fostering the understanding of the world, emotions also direct social action and, if well socialized, make it more effective:“[S]o long as the passions retain this measure of propriety, and are effectual to animate the mind to its proper exertions, it is unnecessary to observe that they are no more than the purpose of nature seems to require; for, even if a person could, without any emotion, ward off the dangers of his country or his friend, we think it becoming, that the energy of his affection should be in due proportion with the occasion on which it is felt” (Ferguson, 1792, p. 128–129). Far from playing a disturbing role with respect to reason, therefore, emotions steer knowledge and action by shaping individual identity and facilitating social interaction. They are present in social interaction, and thus in the making of society, certifying the Fergusonian insight concerning the centrality of the social bond. As was already the case with Smith's reflections on sympathy (Forman-Barzilai, 2009; Weinstein, 2009), particularly in the light of a neurosociological perspective (Özler and Gabrinetti, 2018), Ferguson's idea of rationality can be defined as ‘emotional' as it considers empathy to be helpful to reason, seen as a human faculty oriented toward the solution of social problems. The Fergusonian reason, as Berbalet points out, is a synthesis of different human faculties, including emotional ones, since intellectual liveliness often goes hand in hand with feelings of the heart (Barbalet, 2004, p. 45–61). They participate in the shaping processes of society and confirm Ferguson's insight regarding the multidimensionality of the subjectivity and the centrality of the social bond. The Scottish scholar's social theory, therefore, values emotions as a carrier of knowledge, as a factor of socialization and as a support for social morality.

## 5. Concluding remarks

Ferguson's interest for civil society developed in his most important work shows the author's closeness to the cultural context of his time precisely at the juncture between the emergence of a sociological approach focused on civil society's social relations and the pioneering interest in a discussion of the social role of emotions. Ferguson's vision of history as a contingent outcome of social interaction and its transitory stabilization envisages a sociological criterion to be used in understanding the processes characterizing rising modernity, while the focus on the intersubjective convergence of reasons and feelings outlines a field of research that highlights the analytical relevance of empathy as currently discussed by social sciences.

This Scottish Enlightenment legacy, therefore, is expressed in Ferguson's thought both in the analysis of social processes and in the understanding of everyday interaction. After all, the Scottish Enlightenment considers the study of social interaction as the necessary starting point to understand society and, at the same time, develops a conception of modern subjectivity whose complexity brings with it an important recognition of the individual emotional sphere (Dwyer, 1998). The Enlightenment insight regarding the propensity to social life that characterizes human beings, together with the observation that individuals yearn for mutual recognition, consolidates a sociological view that leaves great space for the emotional implications of social relations. Thus, the meaning subjectively attributed to actions is also discussed from the affective point of view while the reproduction of social norms and moral values leads the Scottish Enlightenment to emphasize the importance of love for community as a factor of social integration. All these issues allow for a critical assessment of Adam Ferguson's thought that suggests its centrality to a reconstruction of the relationship between social theory and emotions. The awareness of the affective dimension of human behavior, as well as the recognition of the contribution that emotions ensure to social interaction, indeed, has encouraged a renewal of social research able to grasp the limits of rationalism and the potential of empathy as a support of sociality. The Scottish sociologist, after all, develops his analysis in relation both to human nature and social processes.

It is possible to consider Ferguson's controversy toward the Mandevillian critique of morality as a good exemplification of the way in which Ferguson uses emotions as an analytical tool to interpret individual expectations. When Mandeville asserts that moral criteria never help to direct the human action, and specifies that those who stigmatize self-interested behavior do so on the basis of equally self-interested criteria, albeit cloaked in ethical considerations, Ferguson focuses his attention on the indignation at this supposed hypocrisy, attributing it to an emotion felt by Mandeville on the basis of his personal moral values, and thus sees it as a glaring denial of the belief that individuals act only on their own interest (Ferguson, 1819, p. 60). Mandeville himself, therefore, would not appear to be exempt from thinking and acting on the basis of emotional dynamics that, as is often the case, lead to moral stances. It is precisely the emotional interpretation of Mandeville's approach and his indignation, that allows Ferguson to prove the fallacy of utilitarianism and to bring into the scientific debate a complex vision of human nature, in which emotions condition morality by playing a leading role in social life. This is all the more relevant insofar as Smith himself, although so attentive to the role of sympathy in social life, opposes Mandevillian arguments with a morally superabundant reflection in which the weight of Stoicism hangs over social analysis.

*An Essay on the History of Civil Society* offers the opportunity to effectively exemplify the way in which Ferguson uses emotions as a useful analytical tool in understanding more general social processes. Although civil society requires peace (Ferrarotti, 1984, p. 8–13), he discusses about war as a recurring historical phenomenon emphasizing its passionate rather than self-interested dimension, as well as the inevitable implications of a conflict of values based on different cultures. Furthermore, certain conflictual dynamics, not only of an immediately warlike nature, would be easily explained in terms of a linear rational utilitarianism whereas “[…] were there no angry passions of different sort, the animosities which attend an opposition of interest, should bear proportion to the supposed value of the subject” (Ferguson, 1819, p. 39); however, the constant disproportion of conflict dynamics proves precisely the opposite, namely the existence of a significant relationship between emotions and individual behavior. War can only be understood as a dimension of social life interconnected with human nature, its moral density and its historical declinations, as far as fight represents a reason for human action that shapes every sphere of social interaction (Hill, 2001), being in itself a further confirmation of the social role of the emotions.

Ultimately, the most significant part of Ferguson's legacy derives from his ability to decline the themes of the Scottish Enlightenment by systematizing their sociological insights and shaping them into a vision of society in which the traits of nascent modernity as well as their emotional dimension find their place. In particular, therefore, it is also from the consideration of the emotions as independent variables of society that “[…] he gives us a proper theory of social change and equilibrium that represents a significant break with both traditional historiographies and contractarian myths of the origins of social institutions and order” (Hill, 2006, p. 8; also Hill, 1996). In this way, Ferguson offers original support to a sociological analysis of modern society particularly dependent on social history, social interaction, and the complexity of human nature.

## Author contributions

The author confirms being the sole contributor of this work and has approved it for publication.
